# Temperature affects the host hematological and cytokine response following experimental ranavirus infection in red-eared sliders *(Trachemys scripta elegans)*

**DOI:** 10.1371/journal.pone.0241414

**Published:** 2020-10-29

**Authors:** Jeremy M. Rayl, Matthew C. Allender

**Affiliations:** Wildlife Epidemiology Laboratory, College of Veterinary Medicine at University of Illinois, Urbana-Champaign, Illinois, United States of America; Universidad Miguel Hernández de Elche, SPAIN

## Abstract

Pathogen-host interactions are important components of epidemiological research, but are scarcely investigated in chelonians. Red-eared sliders (*Trachemys scripta elegans*), are recognized as a model for frog virus-3 infection (FV3), a ranavirus in the family Iridoviridae that infects multiple classes of ectothermic vertebrates. Previous challenge studies observed differences in disease outcome based on environmental temperature in this species, but the host response was minimally evaluated. We challenged red-eared sliders with an FV3-like ranavirus at both 28°C and 22°C. We monitored several host response variables for 30 days, including: survival (binary outcome and duration), clinical signs, total and differential leukocytes, and select cytokine transcription in the buffy coat (IL-1β, TNFα, IFYg, IL-10). After 30 days, 17% of challenged turtles survived at 28°C (Median survival time [MST]: 15 days, range: 10–30 days) and 50% survived (MST: 28.5 days, range: 23–30 days) at 22°C (range 23–30 days). The most common clinical signs were injection site swelling, palpebral swelling, and lethargy. The heterophil/lymphocyte ratio at 22°C and interleukin-1 beta (IL1β) transcription at both 22°C and 28°C were significantly greater on days 9, 16, and 23 in FV3 challenged groups. Tumor necrosis factor alpha and interleukin-10 were transcribed at detectable levels, but did not display significant differences in mean relative transcription quantity over time. Overall, evidence indicates an over-robust immune response leading to death in the challenged turtles. FV3 remains a risk for captive and free-ranging chelonian populations, and insight to host/pathogen interaction through this model helps to elucidate the timing and intensity of the host response that contribute to mortality.

## Introduction

*Ranavirus*, a genus in the family Iridoviridae, contains double-stranded DNA viruses that can infect multiple classes of ectothermic vertebrates [1, 2], frequently resulting in epidemics characterized by high morbidity and mortality [3]. Recognized as a reportable disease in amphibians by the World Organization for Animal Health (OIE), ranaviral disease threatens the biodiversity of amphibian populations worldwide [4]. Certain threatened species demonstrate greater susceptibility compared to more common species [5], indicating conservation concerns which may require species- and season-specific management to account for immune differences between ectotherms.

Frog virus 3-like ranavirus (FV3) is the type species of the genus *Ranavirus* and has a diverse host and geographic range [3]. Frog virus 3 growth is inhibited above 32°C *in vitro*, and species and temporal detection of this virus is often influenced by temperature [6]. Adult red-eared sliders (*Trachemys scripta elegans*, RES) [7] and larval tiger salamanders (*Ambystoma tigrinum*) [8] show lower survival times at colder temperatures. Conversely, higher survival at colder temperatures were observed in largemouth bass [9], larval wood frogs (*Lithobates sylvatica*), larval Cope’s gray tree frogs (*Hyla chrysoscelis*), larval green frogs (*Lithobates clamitans*), larval spotted salamanders (*Ambystoma maculatum*) [10], as well as four chelonian species including juvenile red-eared sliders, Mississippi map turtles (*Graptemys pseudogeographica kohnii*), false map turtles (*Graptemys pseudogeographica*), and eastern river cooters (*Pseudemys cocinna cocinna*) [11]. In the last decade, ranaviral disease has been linked as a cause or contributing factor in multiple mortality events in both free-ranging and captive chelonian populations [12–15]. The additive effects of altered landscapes, human activities (food and pet trades), and disease (ranavirus), are contributing to morbidity and mortality events threatening long-lived and slow-developing species, such as turtles [16, 17]. The host response to the pathogen has largely been ignored in turtles and may be affected by these and other intrinsic and extrinsic factors, thus defining the host-pathogen interaction across a range of environmental temperatures is critical for implementing strategies to minimize disease impact on a landscape [18].

The innate immune system of reptiles is largely considered more robust than the acquired host immune response [19] and commonly measured through changes in hematology [20]. Total and differential white blood cell counts and the heterophil/lymphocyte ratio have been recognized as useful markers among vertebrates for the estimation of infection/inflammation or stress response [21, 22]. Changes based on temperature, age, season, and reproductive status impact these parameters in several reptile species [23]. Cytokines are transient signaling molecules that play a prominent role in innate immunity [24], and have been analyzed through characterization of mRNA transcription analysis in other vertebrate species. In manatees [25] and ferrets [26], cytokine mRNA transcription analysis has been used to determine the host response to a generalized diseased state, and specifically in the model species *Xenopus laevis* following ranavirus infection [27–30]. The cytokine response to ranavirus infection has yet to be investigated for chelonians.

For this study, we used recently developed assays for chelonians [31] to measure cytokine mRNA transcription in conjunction with the hematologic response following experimental ranavirus exposure. Our goal was to characterize host factors that impact survival following an experimental FV3 challenge. Specifically, we hypothesized there will be an increase in the heterophil/lymphocyte ratio, increased transcription of the pro-inflammatory cytokines interleukin-1 beta (IL1β) and tumor necrosis factor alpha (TNFα), and a decrease in the anti-inflammatory cytokine interleukin-10 (IL10) in response to FV3 infection.

## Materials and methods

### Research animals

All protocols were approved under University of Illinois Institutional Animal Care and Use Committee (IACUC Protocol #15225). Animals were obtained from a commercial source and acclimated at the desired temperature for seven days prior to starting the study in matched pairs. Sample size was calculated based on the following *a priori* information: power = 0.8 and an alpha = 0.05, and expected immune response measured by increase of leukocytes and inflammatory cytokines in 80% of the FV3-challenged turtles (N = 6) compared to 5% in the uninfected control group (N = 6) using the Fleiss statistical method for rates and proportions [32]. All challenged turtles were euthanized based on humane end-point criteria established in the approved protocol. Euthanasia was performed within one hour from the time of observation. Surviving turtles were euthanized on day 30, following sample collection.

### Husbandry

Duplicate environmental chambers were maintained, with control and challenged animals housed in separate rooms to avoid accidental exposure. The experiment was conducted sequentially, such that the 28°C experiment occurred first, followed by the 22°C experiment. All turtles were confirmed negative for FV3 in whole blood and oral/cloacal swabs taken at -5 days and -2 days prior to inoculation. Individuals were maintained in 80L tubs with stacked bricks for a basking area out of water. Turtles were fed 2% body weight 4 times per week (Fluker’s Aquatic Turtle Diet, Fluker Farms, Port Allen, LA). Complete water replacements were performed two times per week, with replacement water temperature set room temperature using an infrared thermal camera (FLIR Systems Inc., Wilsonville, OR). Dechlor (Weco Classic Aquarium Products, Long Beach, CA) was added to the water at an appropriate volume according to product directions (1 drop per 1 U.S. gallon, 20 drops per change).

### Virus isolation and inoculation

FV3 was isolated using methods previously described [33]. Viral titers were obtained using serial dilutions in a Terrapene heart cell line (TH-1, DMEM at 27°C) in four technical repeats. Aliquots of 5.0x10^5^ TCID_50_ were frozen at -80°C until the day of inoculation. Inoculated animals were injected intramuscularly in the right forelimb with a single aliquot, while control animals were injected with an equal volume of uninfected TH-1 cell lysate.

### Data collection

Turtles were examined twice daily (morning, evening) for clinical signs related to ranaviral disease, including: ocular discharge, nasal discharge, oral plaques, palpebral swelling, cutaneous abscesses, lethargy, and injection site swelling. All turtles were handled for a maximum of two minutes, and then returned to their enclosure on their basking bricks. Gloves were changed after handling each turtle.

Twice weekly, turtles were weighed, and venous blood (3 ml) was drawn from the subcarapacial sinus. Blood was immediately aliquoted in two separate lithium heparin microtainers (Becton-Dickinson, Franklin Lakes, NJ) and kept on ice until processing 1 hour later.

### Hematology

Packed cell volume (PCV) and total solid (TS) values were performed as previously described [33]. Total white blood cell (WBC) counts were performed using the avian leukopet stain (Vetlab Supply Inc., Palmetto Bay, FL). One-hundred cell count differentials were performed by a single researcher (JMR) from blood smears stained using a modified Wright-Giemsa stain (HEMA III, Fisher Scientific, Waltham, MA).

### FV3 quantification

DNA was extracted from whole blood and oral/cloacal swabs using a commercial kit according to the manufacturer’s protocol (Qiagen DNEasy, Valencia, CA). After extraction, DNA samples were stored at -20°C until analyzed using qPCR [34].

### Cytokine transcription

Briefly, 50 μl of whole heparinized blood was loaded into two plastic microhematocrit tubes (Drummond Scientific Company, Broomall, PA) and centrifuged at 10,000rpm x 5 minutes. Microhematocrit tubes were then cut with a sterile blade to isolate the buffy coat as previously described [35]. The cut portion was placed directly in approximately 60μL of RNALater (Ambion Inc., Foster City, CA), and stored at -20°C until RNA extraction. RNA was extracted using a commercial kit according to the manufacturer’s recommendations (Qiagen RNEasy, Valencia, CA). RNA samples were analyzed using spectrophotometry (NanoDrop 1000, Thermo Fisher) to quantify concentration and purity, then stored at -80°C. Reverse transcription was performed using the Quantitect Reverse Transcription kit (Qiagen, Valencia, CA) following manufacturer’s instructions. The cDNA was diluted with sterile dH_2_0 at 1:40 volume yielding approximately 50ng/μL. All diluted cDNA samples were assayed using a multiplex TaqMan qPCR (qPCR, Biomark HD, Fluidigm, South San Francisco, California, USA) targeting gene transcripts of IL-1β, TNFα, IL-10, and RES beta actin [31] (Table 1). Ten-fold standard dilution series (106–10^0^) of synthesized plasmids containing target gene segments for each cytokine were assayed on each plate.

**Table 1:** Hydrolysis primer-probes used to quantify cytokine mRNA expression in red-eared sliders (*Trachemys scripta elegans*).

Name (NCBI sequence)	Primer-Probe
Beta actin*(MH195268)	F: TGGCCATCTCCTGTTCGAA
	R: GGAAATTGTACGTGACATAAAGGAAA
	P: CCAGAGCAACGTAGCAC
IL1β*(MH195270)	F: GCCCTTCACGGACGATGAT
	R: TCGAATGAGATGGTCTCGAAGA
	P: TGAGGAGCATCTTCGACA
TNFα*(MH195272)	F: CTTTGGGATCCTGGCTGATC
	R: CTTCGCTCTGCTGCATTTCA
	P: AGGGCCCCCCGTTT
IL10*(XM_005306473.1)	F: GCTGCACAAAACTCGCCAAT
	R: GCAATCCGCAGGTCTTTGA
	P: TCCTGCCCCTTCGG

National Center for Biotechnology Information (NCBI) GenBank references are in parentheses. F = forward, R = reverse, P = probe, IL1β = interleukin 1 beta, TNFα = tumor necrosis factor alpha, IL10 = interleukin 10.

*From: Rayl JM, Wellehan Jr. JFX, Bunick D, Allender MC. Development of reverse-transcriptase quantitative PCR assays for the detection of the cytokines IL-1β, TNF-α, and IL-10 in chelonians. Cytokine. 2019;119:16–23.

### Post-mortem samples

Necropsies were performed the day of death. Liver and spleen samples (5mm^3^) were collected for DNA extraction and subsequent FV3 quantification. Additional samples of liver and spleen (5mm^3^) were obtained for each turtle, placed in 500μL RNALater (Ambion), stored at 4°C for a minimum of 24 hours, and then maintained at -80°C freezer until processing. The tissue was disrupted mechanically using a sterile scalpel blade, followed by lysis using sterile glass disruption beads and vortex (10 minutes). RNA was then extracted using the same methods as described above. All samples were transferred to a -20°C freezer until assayed.

### Statistical analysis

Parameter distributions were visually analyzed using ggplot2 [36] and ggpubr [37]. A Kaplan-Meier survival curve was created for each of the 28°C and 22°C challenge groups. For the survival models, fixed effects included group (control, challenge) and room temperature (28°C, 22°C) with outcome as median survival time (MST). Biologically relevant random effects included weight, total white blood cells, total heterophils, total lymphocytes, heterophil/lymphocyte (HL) ratio, total monocytes, total eosinophils, total basophils, packed cell volume (PCV), total solids (TS), cloacal/oral swab (COS) FV3 quantities (ng/μL), whole blood (WB) FV3 quantities (ng/μL), and cytokine mRNA transcription relative to beta actin levels in buffy coat samples. The parameters were evaluated using interaction plots with day and group as fixed effects followed by multivariate evaluation using repeated measures ANOVA. Individual turtles were treated as a constant error term. These models were then compared to generalized linear mixed models treating the individual as a random effect to account for dependent, repeated samples. Autoregressive heterogeneous variance was used as the variance-covariance structure for each model. For variables of interest identified by the ANOVAs and generalized linear mixed models, Kruskal-Wallis tests were used to compare predictor variables within the following groups: Control 28°C, Challenge 28°C, Control 22°C, and Challenge 22°C, as well as to compare between days post-inoculation. Finally, the nine most relevant models were selected for comparison using Akaike’s information criterion. All analyses were computed using RStudio [38]. Survival analyses were performed using Cox proportional hazard models within the ‘survival’ package [39] and AIC analyses were performed using the ‘AICmodavg’ package [40].

## Results

**Fig 1:**
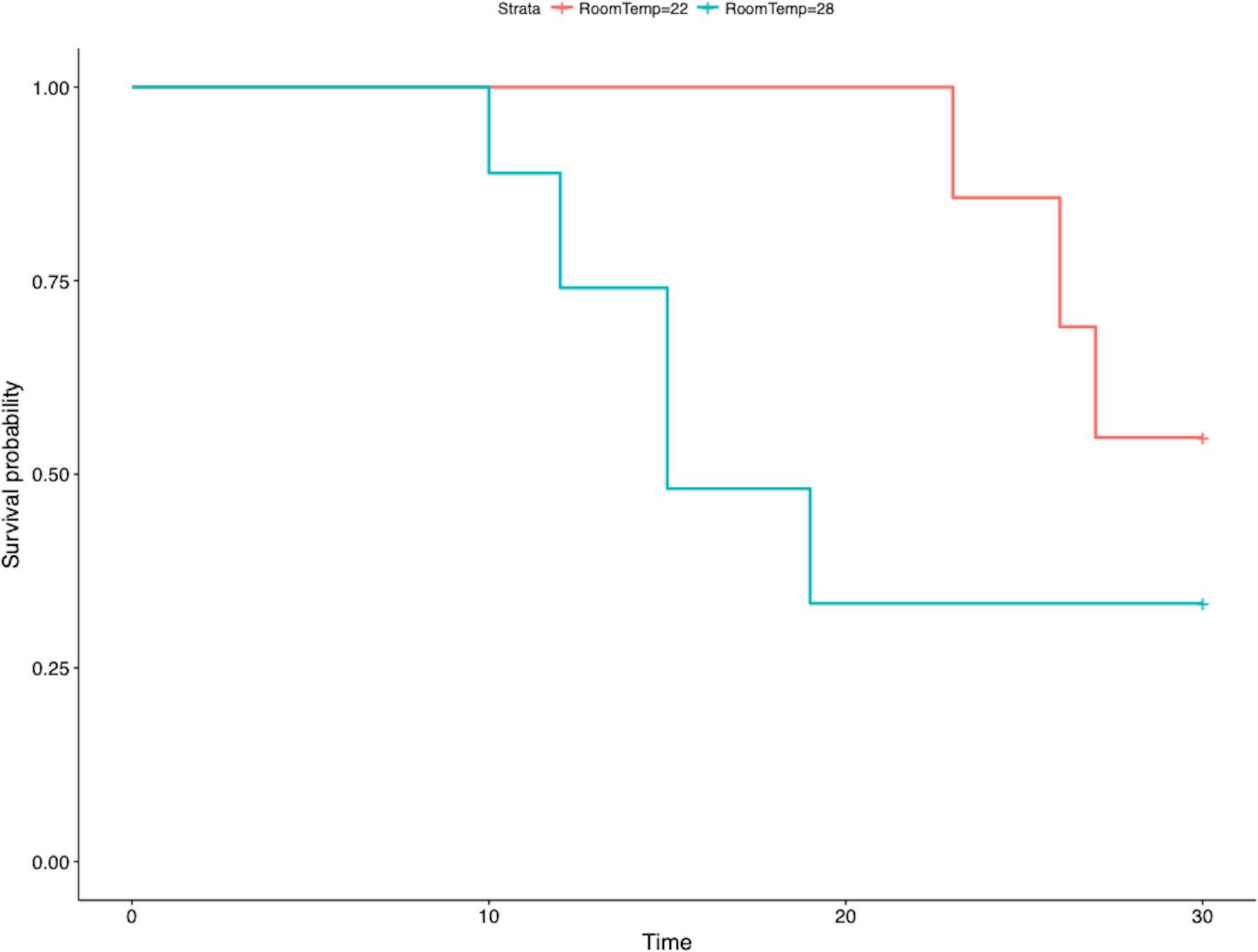
Median survival times for red-eared sliders (*Trachemys scripta elegans*) experimentally challenged with frog virus 3-like ranavirus at 28°C (red) and 22°C (blue) in a 30-day evaluation period.

All turtles were negative for FV3 at day -5 and day -2 in whole blood and oral/cloacal swabs prior to challenge. Once challenged, turtles tested positive for FV3 in whole blood at least once (became infected), and all control turtles remained negative throughout the study in all samples (remained uninfected). At the end of 30 days, 17% of challenged turtles survived (MST: 15 days, range: 10–30 days) at 28°C (range 10–30 days) and 50% survived (MST: 28.5 days, range: 23–30 days) at 22°C (Fig 1). The quantity of FV3 in COS had the greatest support in explaining survival time (S1 Table), but higher viral quantity was not associated with shorter survival time (S1 and S2 Figs).

### Clinical signs

**Fig 2:**
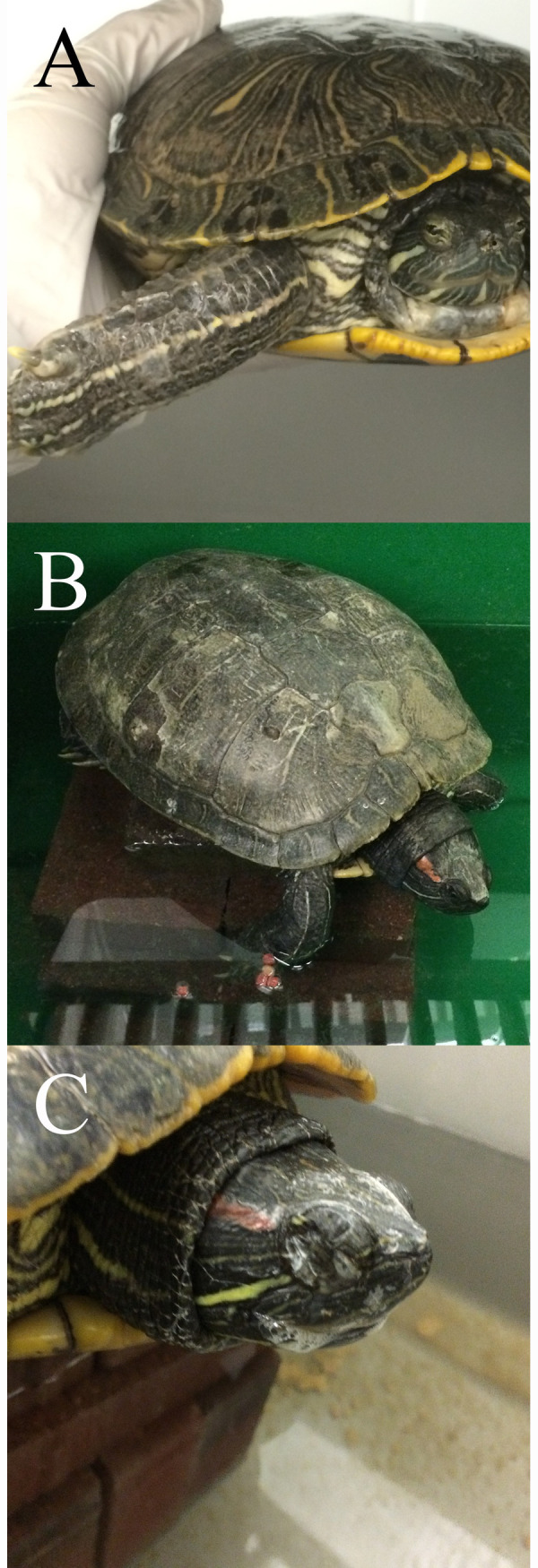
Common clinical signs observed in FV3-challenged red-eared sliders (*Trachemys scripta elegans)* included limb swelling at the injection site (A), lethargy (B), and ocular, nasal, and oral discharge (C).

The most common clinical sign observed was injection site swelling, followed by palpebral swelling and lethargy (Fig 2, Table 2). Ocular discharge, nasal discharge, oral plaques, and cutaneous abscesses occurred inconsistently among challenged turtles (Table 2). A single turtle in the control group at 22°C exhibited ocular discharge and prolonged injection site swelling, but tested negative for FV3.

**Table 2:** Number (percent) of red-eared sliders (*Trachemys scripta elegans*) with each exhibited clinical signs following challenge with frog virus-3 like ranavirus (challenge) or uninfected cell lysate (control) at 28°C and 22°C.

Group	Injection Site Swelling	Palpebral Swelling	Lethargy	Ocular Discharge	Oral Plaque	Cutaneous Abscessation	Nasal Discharge
Control 28°C	2 (33%)	0 (0%)	0 (0%)	0 (0%)	0 (0%)	1 (17%)	0 (0%)
Challenge 28°C	6 (100%)	4 (67%)	6 (100%)	3 (50%)	1 (17%)	0 (0%)	2 (33%)
Control 22°C	4 (67%)	3 (50%)	0 (0%)	1 (17%)	1 (17%)	0 (0%)	1 (17%)
Challenge 22°C	6 (100%)	6 (100%)	6 (100%)	5 (83%)	4 (67%)	2 (33%)	2 (33%)

### Hematology

**Fig 3:**
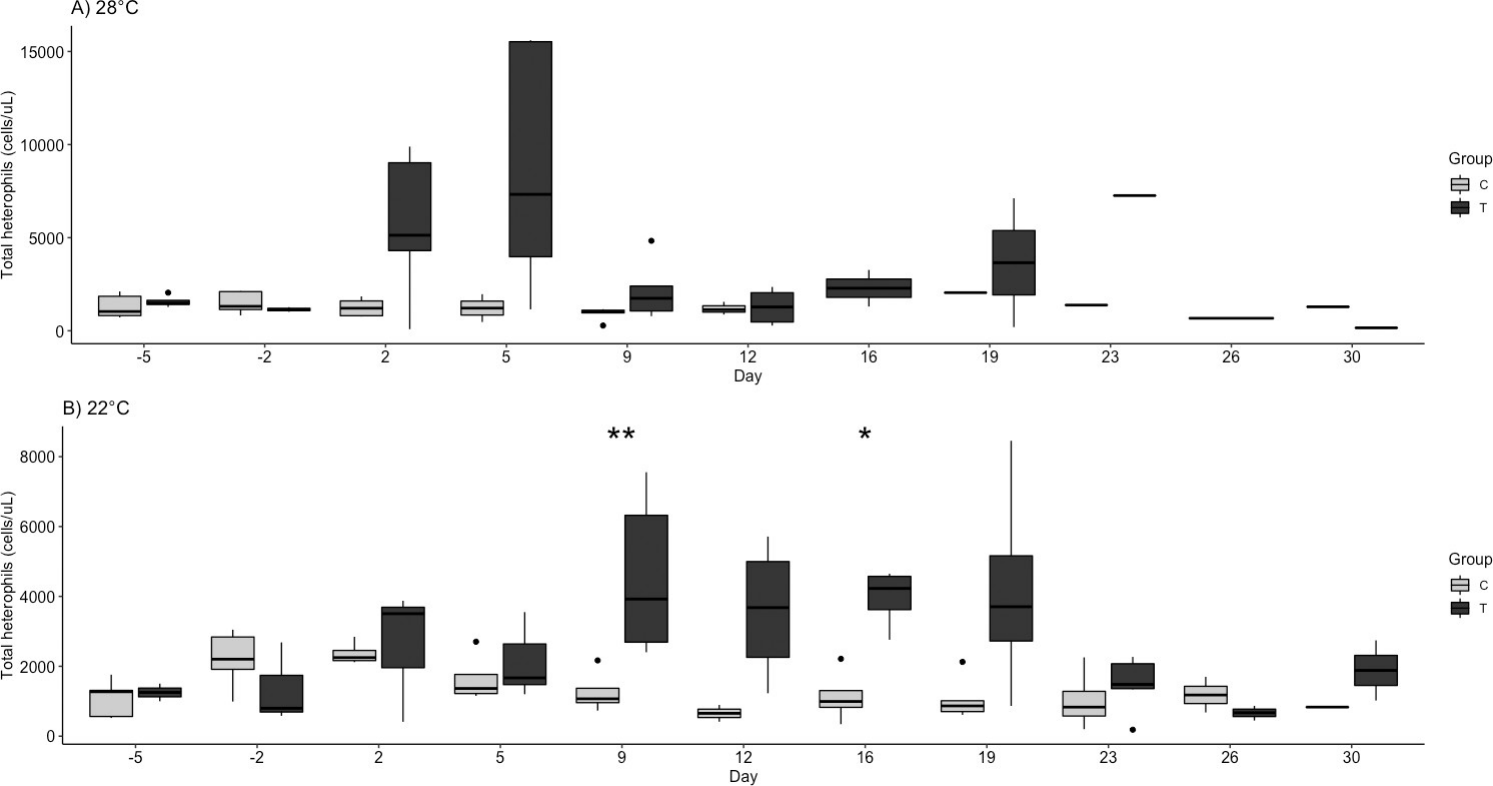
Total heterophil quantity (cells/μl) by group (Control, C and challenge, T) at A) 28°C and B) 22°C following ranavirus challenge of red-eared sliders (*Trachemys scripta elegans*). Outliers are indicated by solid black dots. Significant differences of challenge group compared to control are denoted as follows: * p < 0.05.

**Fig 4:**
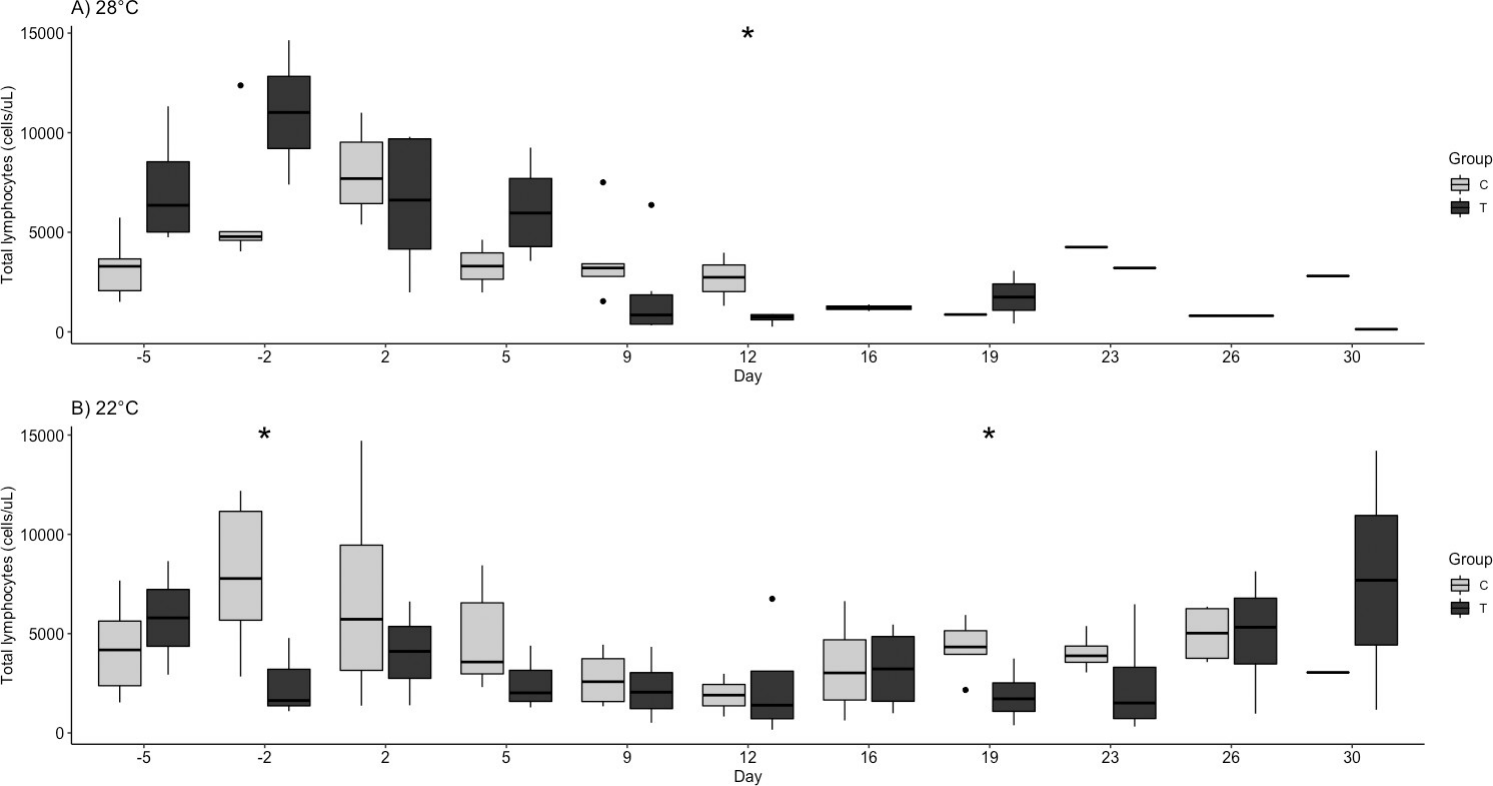
Total lymphocyte quantity (cells/μl) by group (Control, C and Challenge, T) at A) 28°C and B) 22°C following ranavirus challenge of red-eared sliders (*Trachemys scripta elegans*). Outliers are indicated by solid black dots. Significant differences of challenge group compared to control are denoted as follows: * p < 0.05.

**Fig 5:**
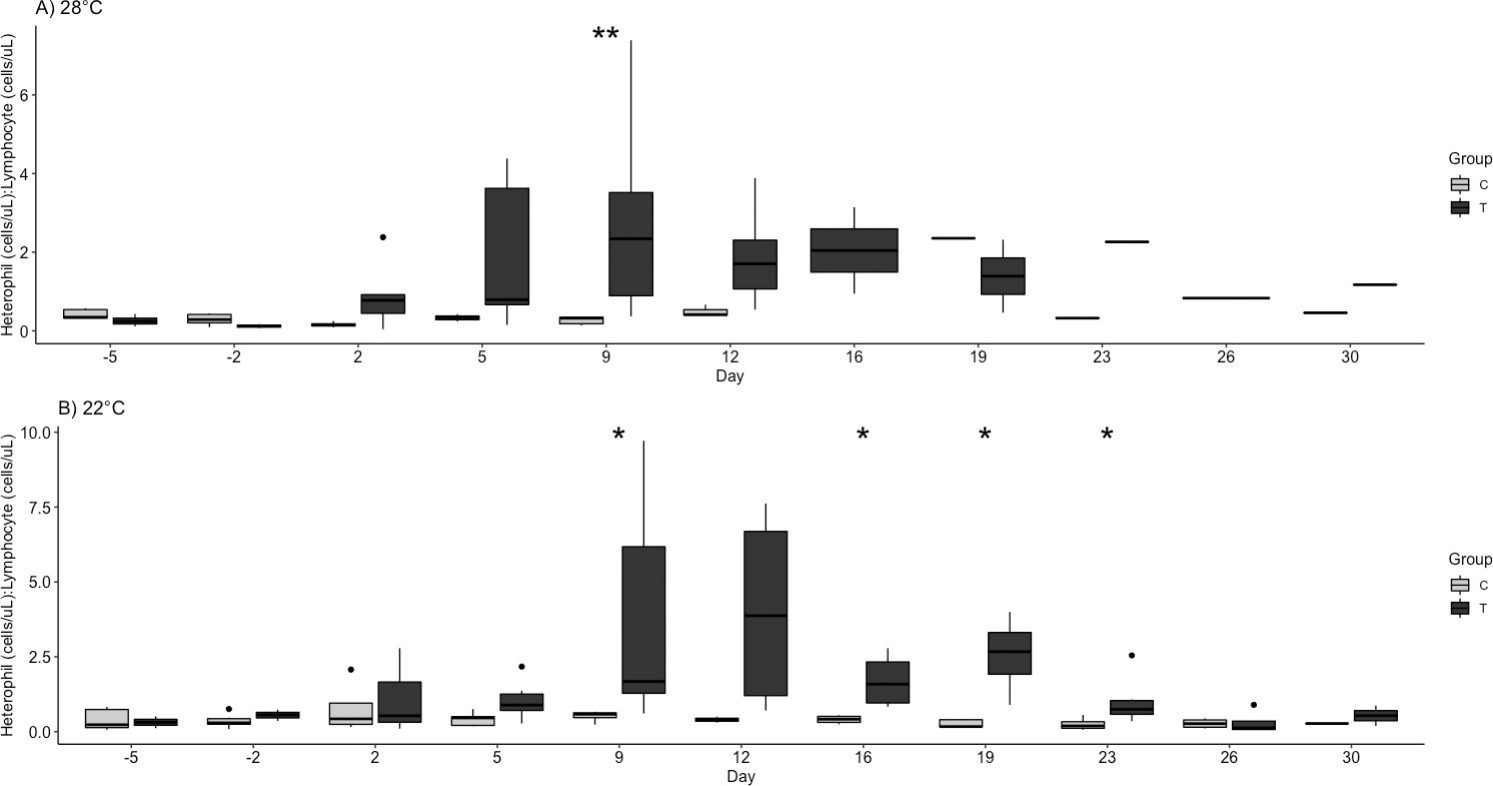
Total heterophil/lymphocyte ratio by group (Control, C and Challenge, T) at A) 28°C and B) 22°C following ranavirus challenge of red-eared sliders (*Trachemys scripta elegans*). Outliers are indicated by solid black dots. Significant differences of challenge group compared to control are denoted as follows: * p < 0.05.

Significant findings are tabulated for hematologic variables (Table 3). The challenged turtles had a non-significant increase in white blood cells, specifically heterophils, on days 2 and 5 at 28°C and a less robust increase at 22°C. By days 9 and 16 at 22°C, the heterophil fraction was significantly higher in the treatment group (p = 0.014, 0.030; Fig 3). Also at 22°C, lymphocytes were significantly lower in the challenge group on day 19 (p = 0.037, Fig 4). Finally, the H:L ratio was significantly higher in the challenge groups at 22°C on days 9, 16, 19, and 23 (p = 0.033, 0.021, 0.014, 0.019; Fig 5).

**Table 3:** Summary of significant differences (Kruskal-Wallis test) across health variables measured in FV3-challenge red-eared sliders (*Trachemys scripta elegans*) compared to sham-inoculated turtles at two controlled ambient room temperatures (28°C, 22°C).

Variable	Difference	Day	P-value
Heterophils	Challenge 22°C > Control 22°C	9	0.014
		16	0.030
Lymphocytes	Challenge	5 > 9	0.018
	Challenge < Control	19	0.037
Heterophil: Lymphocyte	Challenge > Control	9	0.033
		16	0.021
		19	0.014
		23	0.019
Interleukin 1-beta	Challenge 22°C > Control 22°C	5	0.014
		9	0.025
		19	0.020
		23	0.043
		26	0.030
	28°C > 22°C	2	0.024
		5	0.001
		9	0.015
	Liver 28°C Challenge > Control		0.005
	Liver 22°C Challenge > Control		0.005
Interleukin 10	No differences		
Tumor necrosis factor alpha	No differences		

### Molecular assays

**Fig 6:**
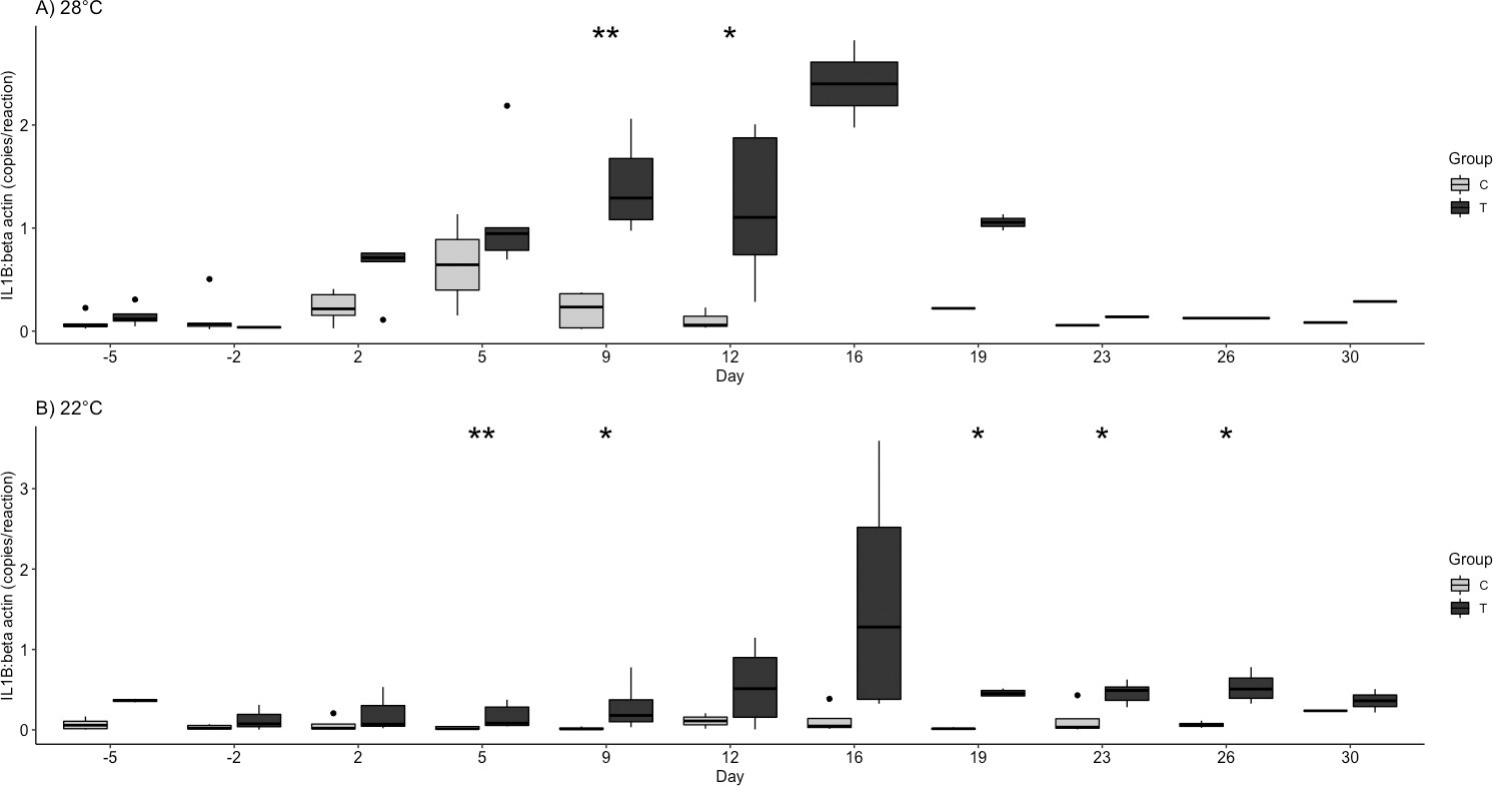
Interleukin-1 beta (IL1B β) relative mRNA transcription in buffy coat by group (Control, C and Challenge, T) at A) 28°C and B) 22°C following ranavirus challenge of red-eared sliders (*Trachemys scripta elegans*). Outliers are indicated by solid black dots. Significant differences of challenge group compared to control are denoted as follows: * p < 0.05, ** p < 0.01.

Final cloacal/oral swabs and blood samples were taken prior to euthanasia; FV3 quantities were higher at 22°C compared to 28°C. Significant findings are tabulated for cytokine variables (Table 3). Relative transcription of IL1β was significantly higher in the challenge group at 22°C on days 5, 9, 19, 23, and 26 (Fig 6). Relative transcription of IL1β was significantly higher in the control and challenge groups at 28°C compared to both groups at 22°C on day 2 (p = 0.024), day 5 (p = 0.001), and day 9 (p = 0.015). For the 28°C groups, the increase in relative IL1β transcription occurred sooner and to a greater extent compared to the 22°C groups; the challenge group at 28°C continued to rise while the control group at 28°C peaked at day 5 and then decreased (Fig 6). IL1β transcription was the highest on days 12 and 16 post-infection in the challenged turtles at both temperatures. Transcripts of TNFα and IL-10 were observed at detectable levels in the isolated buffy coat, but no significant differences were observed between challenge and control groups at 28°C or 22°C. Among the other cytokines measured in this study, none showed significant differences in relative transcription between groups or temperatures.

### Post-mortem samples

**Fig 7:**
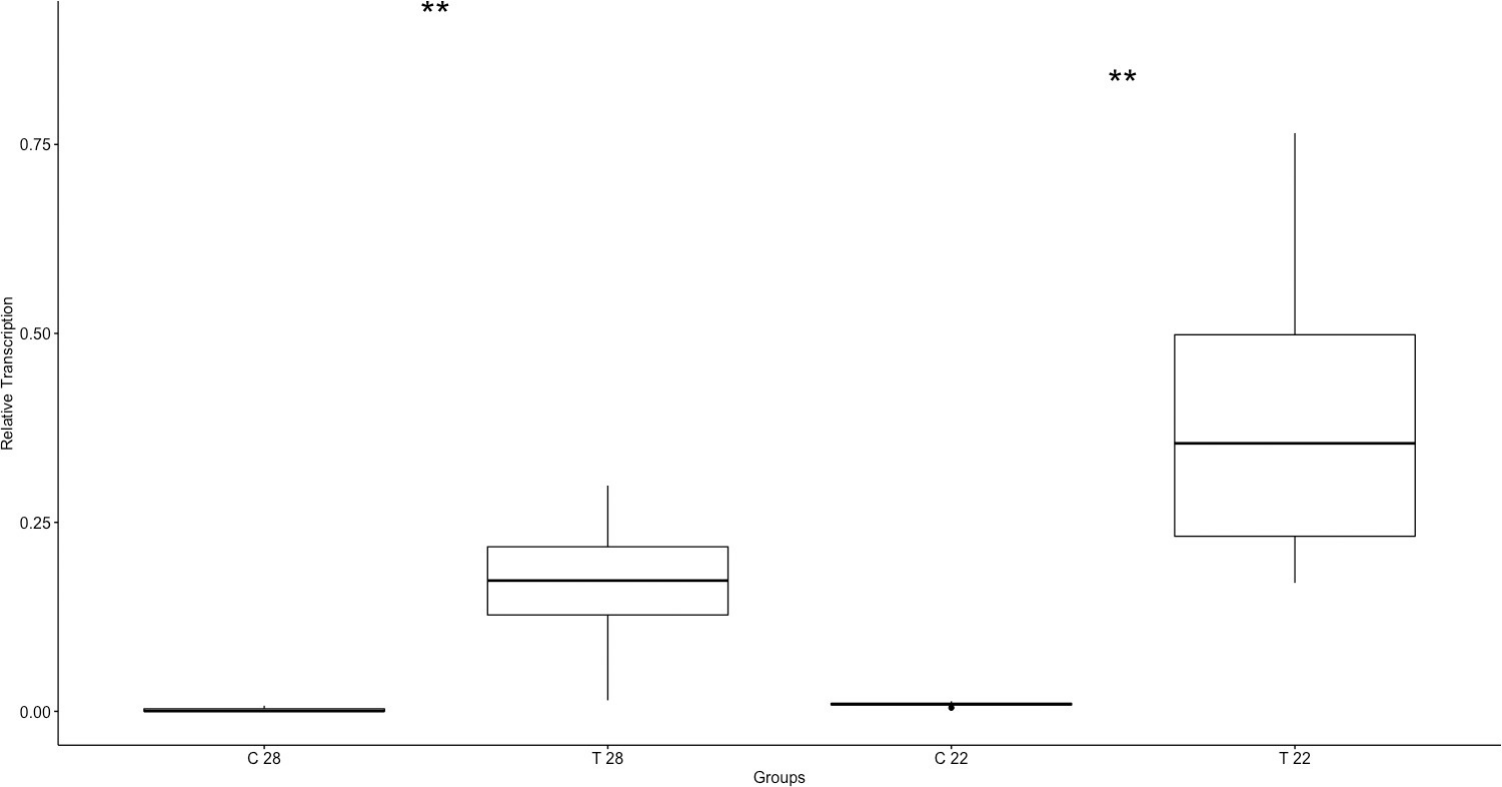
Interleukin-1 beta (IL1β) relative mRNA transcription in liver tissue by group [28°C control (C 28) and challenge (T 28), 22°C control (C 22) and challenge (T 22)] following ranavirus challenge of red-eared sliders (*Trachemys scripta elegans*). Significant differences (**p < 0.01) between challenge and control groups.

IL1β had significantly greater transcription in the liver of individuals in the challenge groups at both temperatures (28°C p = 0.005, 22°C p = 0.005, Fig 7). No other cytokines detected in tissues showed significant differences in relative transcription between challenge and control groups of either temperature.

## Discussion

We set out to monitor the host response in red-eared sliders experimentally infected with FV3-like ranavirus. Turtles challenged with FV3 were observed with measurable and significantly different hematologic (heterophils, lymphocytes, HL ratio) findings and cytokine (IL1β) quantities at two different environmental temperatures indicative of infection. The innate immune response, including cytokine signaling, is considered the primary response to infection in ectotherms, and likely is the response to FV3 [19]. A robust cytokine response has been used to predict the outcome of viral diseases in other vertebrate species, specifically in ferrets infected with morbillivirus [41] and *Xenopus* exposed to FV3 [28]. We observed differences in IL1β transcription between challenged and control groups as well as between temperatures. One of the most intense responses included a relative increase in IL1β transcription, which was observed earlier in the 28°C treatment group. The sharp increase of IL1β transcription at day 12 post-inoculation combined with an increase in clinical signs and mortality at the warmer temperature is consistent with excessive cytokine signaling, in which the host immune response is strong enough to cause self-damage [42]. Most of the turtles in the 28°C temperature were euthanized at the peak IL1β relative transcription levels at days 12 and 16, providing evidence that mortality may have been driven by this exaggerated immune response.

Challenged turtles had a sharp increase in white blood cells, driven mainly by a relative heterophilia on days 2 and 5 at 28°C and a less robust increase at 22°C. This, coupled with relative lymphopenia resulted in an increased HL ratio. The HL ratio is commonly an indicator for increased inflammatory response in birds, reptiles, and amphibians [43, 44]. The ratio was significantly highest just prior to peak IL1β relative transcription levels and follows the timing of increases in white blood cell counts in other reptiles viral infections, specifically in snakes with ferlaviruses between days 4 and 16 post-infection [45]. Thus, the timing of the hematological response has potential to be used to identify viral disease progression and inform ranaviral disease treatment.

Despite relative increases, cytokine transcription alone did not explain survival, and remains a limitation for management decisions. This may be because transcription does not reflect true circulating cytokine concentration or the timing of sampling did not detect key changes that would improve the statistical models. Frog virus 3-like virus outbreaks in the wild have occurred in the relatively hotter months in North America of June and July [13], and mortality is likely influenced by both select host and pathogen factors. While ranavirus replication occurs across a range from 12–32°C with the highest growth at 30°C [6], the host immune response is also increased at the higher end of their preferred optimum temperature zone [19, 46]. Subsequently, it seems reasonable to alternatively conclude that a robust host response contributed to the mortality since fewer viral copies were observed at 28°C compared to the 22°C group.

The TNFα and IL-10 assays had detectable and highly variable transcription, thus no significant associations with outcome of viral quantity were observed in this study. Despite the findings, these cytokines still have the potential to provide insight as biomarkers for disease processes in red-eared sliders and other chelonian species. The timing of sampling may impact the transcription levels that were observed, which were near the limit of detection for the assay. More frequent sampling protocols could yield stronger responses than we observed. Conversely, these cytokines may be transcribed at naturally low levels in red-eared sliders, as was observed in ferrets [26], and more sensitive assays will be needed with larger sample sizes to detect significant changes in transcription.

The development of multifactorial analyses and models from the host data gained in this study, specifically heterophil count, HL ratio, and IL1β relative mRNA transcription were most informative for individual host response. Future studies should investigate the use of a less potent viral dose that may result in a lower mortality and longer duration of infection, potentially providing the opportunity to observe subtle hematologic and cytokine changes that result in lower mortality, thus elucidating mechanisms that might identify treatment options aimed at the innate immune response. Evidence for local cytokine manipulation in rat models shows the potential for influencing disease outcome in the spine [47] and kidneys [48]. However, it remains to be determined if systemic cytokine modification can improve outcome in viral disease, specifically for ranavirus in chelonians. Other treatment options include immune suppression through the use of steroids and anti-inflammatories prior to the peak cytokine signaling events, but additional trials would be necessary to determine the efficacy and appropriate treatment dose and dosage without compromising the productive immune response. Improving the adaptive immune response has been achieved through vaccination in certain amphibians, such as the Chinese giant salamander (*Andrias davidianus*) [49]. These responses have not been observed in reptiles to date, but components of the innate and adaptive immune response including type 1 interferon, major histocompatibility complex, and immunoglobulin M should be evaluated as novel gene transcription targets in chelonians [50].

The chelonian immune system is complex and our approach was able to further characterize the innate immune response to viral infection. Currently, we identified a relative heterophilia, relative lymphopenia, increased HL ratio, and robust IL1β relative mRNA transcription as useful in detection of the chelonian immune response to FV3 infection. Together, with evidence of characteristic FV3 lesions on histopathology including hematopoietic tissue necrosis and fibrinous vasculitis from another study [15], these data suggest that multisystemic inflammation contributes to death in red-eared sliders infected with FV3, though the factors are not statistically significant predictors of mean survival time. Continuing to monitor these and other cytokine variables may inform research into the immune response to other pathogens and in various chelonian species. As techniques and approaches change, it is necessary to review previous models to support the conclusions and find new mechanisms for observed patterns in viral infection. In doing so, we can better understand host-pathogen relationships and make more accurate outcome predictions for disease management in captive and free-ranging chelonians.

## Supporting information

S1 Table AICc table from R analysis of 9 models built based on expected greatest contribution to survival outcome from health parameters of red-eared slider (*Trachemys scripta elegans*) infection with frog virus 3-like virus (FV3). COS = cloacal/oral swab, WB = whole blood, PCV = packed cell volume, TS = total solids, TotWBC = total white blood cells, IL1BRel = relative transcription of interleukin 1-beta.
(DOCX)

S1 Fig Frog virus 3-like virus (FV3) copies per nanogram DNA in whole blood and oral/cloacal (COS) swabs over the 30 day challenge in red-eared sliders (*Trachemys scripta elegans*). Note: One outlier at >60,000 copies/nanogram was excluded from this figure for scale clarity (Day 23, 22°C).
(TIF)

S2 Fig Frog virus 3-like virus (FV3) copies/ng DNA in the liver and spleen of infected red-eared sliders (*Trachemys scripta elegans*) at 22°C and 28°C. (TIF)

S1 Dataset (CSV)

## References

[1] Chinchar VG, Yu KH, Jancovich JK. The molecular biology of frog virus 3 and other iridoviruses infecting cold-blooded vertebrates. Viruses. 2011;1959–1985; 10.3390/v3101959 22069524 PMC3205390

[2] Brenes R, Gray MJ, Waltzek TB, Wilkes RP, Miller DL. Transmission of ranavirus between ectothermic vertebrate hosts. PLOS ONE. 2014; 9(3): e92476 10.1371/journal.pone.0092476 24667325 PMC3965414

[3] Duffus ALJ, Waltzek TB, Stöhr AC, Allender MC, Gotesman M, Whittington RJ, et al Distribution and Host Range of Ranaviruses In: Ranaviruses. Gray MJ and Chinchar VG (eds.). Springer Open 2015;9–57. 10.1007/978-3-319-13755-1_1

[4] Lesbarrères D, Balseiro A, Brunner J, Chinchar VG, Duffus A, Kerby J, et al 2012. Ranavirus: past, present and future. Biol Lett. 2012;8:481–483. 10.1098/rsbl.2011.0951 22048891 PMC3391431

[5] Sutton WB, Gray MJ, Hardman RH, Wilkes RP, Kouba AJ, Miller DL. High susceptibility of the endangered dusky gopher frog to ranavirus. Dis Aquat Organ. 2014;112:9–16. 10.3354/dao02792 25392038

[6] Gravell M and Granoff A. Viruses and renal carcinoma of Rana pipiens: The influence of temperature and host cell on replication of frog polyhedral cytoplasmic deoxyribovirus (PCDV). Virology. 1970;41:596–602. 10.1016/0042-6822(70)90425-3 5528982

[7] Allender MC, Mitchell MA, Torres T, Sekowska J. Pathogenicity of frog-virus 3-like virus in red-eared slider turtles (*Trachemys scripta elegans*) at two environmental temperatures. J Comp Path. 2013;149(2–3): 356–357. 10.1016/j.jcpa.2013.01.007 23582975

[8] Rojas S, Richards K, Jancovich JK, Davidson EW. Influence of temperature on ranavirus infection in larval salamanders *Ambystoma tigrinum*. Dis Aquat Organ. 2005;63:95–100. 10.3354/dao063095 15819423

[9] Grant EC, Philipp DP, Inendino KR, Goldberg TL. Effects of temperature on the susceptibility of largemouth bass to largemouth bass virus. J Aquat Anim Health. 2003;15:215–220.

[10] Brand MD, Hill RD, Brenes R, Chaney JC, Wilkes RP, Grayfer L, et al Water temperature affects susceptibility to ranavirus. Ecohealth. 2016;13:350–359. 10.1007/s10393-016-1120-1 27283058

[11] Allender MC, Barthel A, Rayl JM, Terio K. Experimental transmission of frog-virus 3-like ranavirus in juvenile chelonians *(Trachemys scripta elegans*, *Graptemys pseudogeographica kohnii*, *Graptemys pseudogeographica*, *and Pseudemys concinna)* at two temperatures. J Wild Dis. 2018;54(4);716–725. 10.7589/2017-07-181 29878878

[12] Allender, M.C. 2012. Characterizing the Epidemiology of Ranavirus in North American Chelonians: Diagnosis, Surveillance, Pathogenesis, and Treatment. PhD Dissertation, University of Illinois at Urbana-Champaign.

[13] Adamovicz L, Allender MC, Archer G, Rzadkowska M, Boers K, Phillips C, et al Investigation of multiple mortality events in eastern box turtles (*Terrapene carolina carolina*). PLOS One. 2018 4 5;13(4):e0195617 10.1371/journal.pone.0195617 29621347 PMC5886585

[14] Sim RR, Allender MC, Crawford LK, Wack AN, Murphy KJ, Mankowski JL, et al Ranavirus epizootic in captive Eastern Box Turtles (*Terrapene carolina carolina*) with concurrent herpesvirus and mycoplasma infection: management and monitoring. J Zoo Wildl Med. 2016;47:256–270. 10.1638/2015-0048.1 27010285

[15] Johnson AJ, Pessier AP, Wellehan JFX, Childress A, Norton TM, Stedman NL, et al Ranavirus infection of free-ranging and captive box turtles and tortoises in the United States. J Wildl Dis. 2008;44:851–863. 10.7589/0090-3558-44.4.851 18957641

[16] Gibbons JW, Scott DE, Ryan TJ, Buhlmann KA, Tuberville TD, Metts BS, et al The global decline of reptiles, déjà vu amphibians. BioScience. 2000;50(8):653–666.

[17] Heppell SS. Application of life-history theory and population model analysis to turtle conservation. Copeia. 1998;2:367–375.

[18] Hawley DM, Altizer SM. Disease ecology meets ecological immunology: understanding the links between organismal immunity and infection dynamics in natural populations. Func Ecol. 2011;25:48–60.

[19] Rios FM, Zimmerman LM. Immunology of Reptiles. *eLS*. 2015;1–7. http://doi.wiley.com/10.1002/9780470015902.a0026260.

[20] Nardini G, Leopardi S, Bielli M. Clinical hematology in reptilian species. Vet Clin Exot Anim. 2013;16:1–30. 10.1016/j.cvex.2012.09.001 23347537

[21] Davis AK, Maney DL, Maerz JC. The use of leukocyte profiles to measure stress in vertebrates: A review for ecologists. Func Ecol. 2008;22:760–772.

[22] Lloyd TC, Allender MC, Archer G, Phillips CA, Byrd J, Moore AR. Modeling hematologic and biochemical parameters with spatiotemporal analysis for the free-ranging Eastern box turtle (*Terrapene carolina carolina*) in Illinois and Tennessee, a potential biosentinel. Ecohealth. 2016;13:467–479. 10.1007/s10393-016-1142-8 27384647

[23] Sykes JM, Klaphake E. Reptile Hematology. Clin Lab Med. 2015;3:661–680. 10.1016/j.cll.2015.05.014 26297412

[24] Bienvenu J, Monneret G, Fabien N, Revillard JP. The clinical usefulness of the measurement of cytokines. Clin Chem Lab Med. 2000;38:267–285. 10.1515/CCLM.2000.040 10928646

[25] Ferrante, J.A. 2014. Characterization of cytokine levels and Trichechid herpesvirus I load in the Florida manatee (*Trichechus manitus latirostris*). PhD Dissertation, University of Florida.

[26] Carolan LA, Butler J, Rockman S, Guarnaccia T, Hurt AC, Reading P, et al TaqMan real time RT-PCR assays for detecting ferret innate and adaptive immune responses. J Virol Methods. 2014;205:38–52. 10.1016/j.jviromet.2014.04.014 24797460 PMC7113642

[27] Morales HD, Abramowitz L, Gertz J, Sowa J, Vogel A, Robert J. Innate immune responses and permissiveness to ranavirus infection of peritoneal leukocytes in the frog *Xenopus laevis*. J Virol. 2010;84:4912–4922. 10.1128/JVI.02486-09 20200236 PMC2863837

[28] De Jesús Andino F, Chen G, Li Z, Grayfer L, Robert J. Susceptibility of *Xenopus laevis* tadpoles to infection by the ranavirus frog-virus 3 correlates with a reduced and delayed innate immune response in comparison with adult frogs. Virology. 2012;432(2):435–443. 10.1016/j.virol.2012.07.001 22819836 PMC3574294

[29] Grayfer L, Andino FDJ, Chen G, Chinchar G V, Robert J. Immune evasion strategies of ranaviruses and innate immune responses to these emerging pathogens. Viruses. 2012;4:1075–1092. 10.3390/v4071075 22852041 PMC3407895

[30] Grayfer L, De Jesús Andino F, Robert J. Prominent amphibian (*Xenopus laevis*) tadpole type III interferon response to the frog virus 3 Ranavirus. J Virol. 2015;89:5072–5082. 10.1128/JVI.00051-15 25717104 PMC4403449

[31] Rayl JM, Wellehan JFX Jr., Bunick D, Allender MC. Development of reverse-transcriptase quantitative PCR assays for the detection of the cytokines IL-1β, TNF-α, and IL-10 in chelonians. Cytokine. 2019;119:16–23. 10.1016/j.cyto.2019.02.011 30856601

[32] Dean AG, Sullivan KM, Soe MM. OpenEpi: Open Source Epidemiologic Statistics for Public Health, Version. www.OpenEpi.com, updated 2013/04/06.

[33] Allender MC, Mitchell MA. Hematologic response to experimental infections of frog virus 3-like virus in ed-eared sliders (*Trachemys scripta elegans*). J Herp Med Surg. 2013;23(1–2):25–31.

[34] Allender MC, Bunick D, Mitchell MA. Development and validation of TaqMan quantitative PCR for detection of frog virus 3-like virus in eastern box turtles (*Terrapene carolina carolina*). J Virol Met. 2013;188:121–125. 10.1016/j.jviromet.2012.12.012 23274753

[35] Yabuki A, Sawa M, Chang H-S, Yamato O. A practical technique for electron microscopy of buffy coats in dogs and cats. Anat Histol Embryol. 2014;44: 317–320. 10.1111/ahe.12143 25181932

[36] Wickham H. ggplot2: Elegant Graphics for Data Analysis. Springer-Verlag New York 2016: ISBN 978-3-319-24277-4; https://ggplot2.tidyverse.org

[37] Kassambara A. 2018. Ggpubr: ‘ggplot2’ Based Publication Ready Plots. R package version 0.1.8. https://cran.r-project.org/package=ggpubr.

[38] R Core Team. 2016. R: A language and environment for statistical computing. R Foundation for Statistical Computing, Vienna, Austria. URL https://www.R-project.org/.

[39] Therneau T. 2015. _A Package for Survival Analysis in S_. version 2.38. https://CRAN.R-project.org/package=survival.

[40] Mazerolle MJ. AICcmodavg: Model selection and multimodel inference based on (Q)AIC(c). R package version 2.2–2. https://cran.r-project.org/package=AICcmodavg.

[41] Svitek N, von Messling V. Early cytokine mRNA expression profiles predict Morbillivirus disease outcome in ferrets. Virology. 2007;362:404–410. 10.1016/j.virol.2007.01.002 17289104 PMC2697062

[42] Tisoncik JR, Korth MJ, Simmons CP, Farrar J, Martin TR, Katze MG. Into the Eye of the Cytokine Storm. Microbiol Mol Biol Rev. 2012;76:16–32. 10.1128/MMBR.05015-11 22390970 PMC3294426

[43] Gross WB and Siegel HS. Evaluation of the heterophil/lymphocyte ratio as a measure of stress in chickens. Avian Dis. 1983;27(4):972–979. 6360120

[44] Goessling JM, Kennedy H, Mendonça MT, Wilson AE. A meta-analysis of plasma corticosterone and heterophil:lymphocyte ratios—is there conservation of physiological stress responses over time? Func Ecol. 2015;29(9):1189–1196.

[45] Neul A, Schrödl W, Marschang RE, Bjick T, Truyen U, von Buttlar H, et al Immunologic responses in corn snakes (*Pantherophis guttatus*) after experimentally induced infection with ferlaviruses. Am J Vet Res. 2017;78(4):482–494. 10.2460/ajvr.78.4.482 28345994

[46] Zimmerman LM, Carter AW, Bowden RM, Vogel LA. Immunocompetence in a long-lived ectothermic vertebrate is temperature dependent but shows no decline in older adults. Fun Ecol. 2017;31(7):1383–1389.

[47] Whitehead KJ, Smith CGS, Delaney S-A, Curnow SJ, Salmon M, Hughes JP, et al Dynamic regulation of spinal pro-inflammatory cytokine release in the rat *in vivo* following peripheral nerve injury. Brain, Behav and Immun. 2010;24(4):569–576. 10.1016/j.bbi.2009.12.007 20035858

[48] Banki E, Degrell P, Kiss P, Kovacs K, Kemeny A, Csanaky K, et al Effect of PACAP treatment on kidney morphology and cytokine expression in rat diabetic nephropathy. Peptides. 2013;42:125–130. 10.1016/j.peptides.2013.02.002 23416022

[49] Chen ZY, Li T, Gao XC, Wang CF, Zhang QY. Protective immunity induced by DNA vaccination against ranavirus infection in Chinese giant salamander *Andrias davidianus*. Viruses. 2018;10;52 10.3390/v10020052 29364850 PMC5850359

[50] Ke F and Zhang QY. Aquatic animal viruses mediated immune evasion in their host. Fish and Shellfish Immunology. 2019;86;1096–1105. 10.1016/j.fsi.2018.12.027 30557608

