# Supplementary material for: Temperature affects the host hematological and cytokine response following experimental ranavirus infection in red-eared sliders (Trachemys scripta elegans)
Source: PLoS One. 2020 Oct 29;15(10):e0241414. doi: 10.1371/journal.pone.0241414 (PMC7595395; doi:10.1371/journal.pone.0241414)
Supplement: S1 Table — COS = cloacal/oral swab, WB = whole blood, PCV = packed cell volume, TS = total solids, TotWBC = total white blood cells, IL1BRel = relative transcription of interleukin 1-beta. (DOCX) [file pone.0241414.s001.docx]

**S1 Table**. AICc table from R analysis of 9 models built based on expected greatest contribution to survival outcome from health parameters of red-eared slider (*Trachemys scripta elegans*) infection with frog virus 3-like virus (FV3). COS = cloacal/oral swab, WB = whole blood, PCV = packed cell volume, TS = total solids, TotWBC = total white blood cells, IL1BRel = relative transcription of interleukin 1-beta.

|  | **K** | **AICc** | **Δ AICc** | **AICc Weight** | **Cumulative Weight** | **Least Likelihood** |
| --- | --- | --- | --- | --- | --- | --- |
| **COS FV3** | 2 | 148.05 | 0.00 | 0.74 | 0.74 | -71.94 |
| **WB FV3 + COS FV3** | 3 | 150.20 | 2.15 | 0.25 | 1 | -71.93 |
| **WB FV3** | 2 | 160.31 | 12.26 | 0 | 1 | -78.07 |
| **Heterophil:**  **Lymphocyte** | 2 | 162.38 | 14.33 | 0 | 1 | -79.11 |
| **IL1BRel** | 2 | 162.49 | 14.45 | 0 | 1 | -79.17 |
| **Null** | 1 | 163.77 | 15.72 | 0 | 1 | -80.86 |
| **PCV** | 2 | 165.89 | 17.84 | 0 | 1 | -80.86 |
| **WBC** | 2 | 165.94 | 17.89 | 0 | 1 | -80.86 |
| **Global** | 10 | 168.02 | 19.97 | 0 | 1 | -72.29 |
| **PCV + TS + TotWBC** | 4 | 170.74 | 22.70 | 0 | 1 | -81.10 |
| **TS** | 2 | 173.72 | 25.28 | 0 | 1 | -84.58 |
