# Supplementary figures and images for: Temperature affects the host hematological and cytokine response following experimental ranavirus infection in red-eared sliders (Trachemys scripta elegans)
Source: PLoS One. 2020 Oct 29;15(10):e0241414. doi: 10.1371/journal.pone.0241414 (PMC7595395; doi:10.1371/journal.pone.0241414)

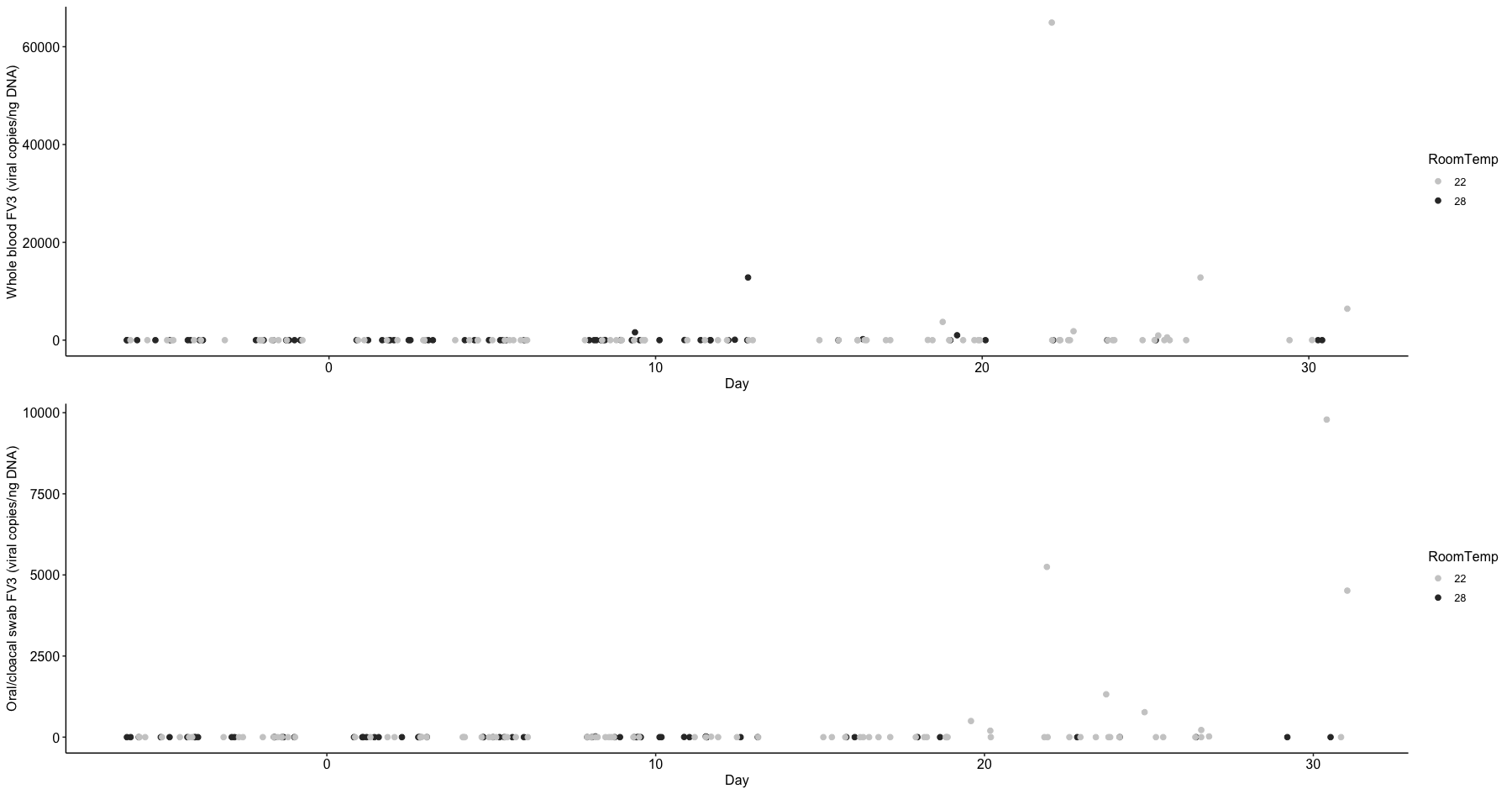

Supplement: S1 Fig — Note: One outlier at >60,000 copies/nanogram was excluded from this figure for scale clarity (Day 23, 22°C). (TIF) [file pone.0241414.s002.tif]

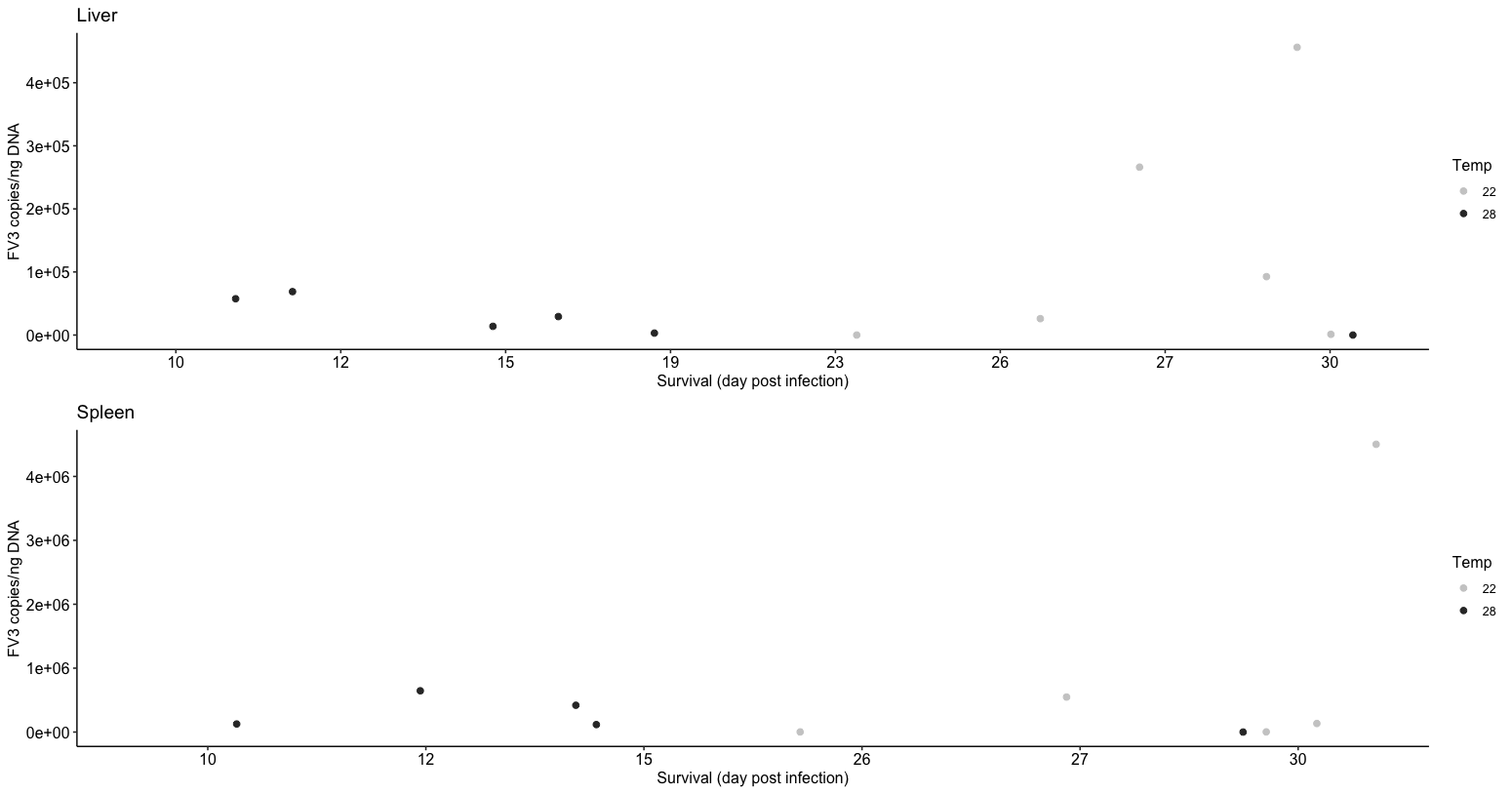

Supplement: S2 Fig — (TIF) [file pone.0241414.s003.tif]
